# After a Century of Research into Environmental Mutagens and Carcinogens, Where Do We Stand?

**DOI:** 10.3390/ijerph20021040

**Published:** 2023-01-06

**Authors:** João D. Vitorino, Pedro M. Costa

**Affiliations:** Associate Laboratory i4HB—Institute for Health and Bioeconomy, NOVA School of Science and Technology, NOVA University of Lisbon, 2829-516 Caparica, Portugal

**Keywords:** neoplasia, cancer, pollution, contamination, genotoxicants, occupational exposure, risk assessment, biomedicine

## Abstract

Cancer is one of the longest-known human diseases, yet only in recent times have we begun to perceive that the percentage of neoplasms caused by environmental factors, lifestyle and chemicals, is likely underestimated. The first medical reports associating cancer with pollutants like tars appeared by the early 20th century, but despite initial evidence relating oncogenesis and chromosomal alterations, only after the structure of DNA had been elucidated in the 1950s have genetic disorders been fully perceived as cause. This led to a growing interest in genotoxic and mutagenic pollutants. Even though we are now familiar with a range of environmental carcinogens spanning between aromatic hydrocarbons and asbestos to radionuclides and forms of carbon nanomaterials, establishing causal networks between pollutants and cancer remains cumbersome. In most part, this is due to the complexity of toxicant matrices, unknown modes-of-action of chemicals or their mixtures, the widening array of novel pollutants plus difficulties in subtracting background effects from true aetiology of disease. Recent advances in analytical chemistry, high-throughput toxicology, next-generation sequencing, computational biology and databases that allocate whole normal and cancer genomes, all indicate that we are on the verge of a new age of research into mechanistic ‘oncotoxicology’, but how can it impact risk assessment and prevention?

## 1. Introduction

Cancer is one of the longest-known human diseases. The first scientific descriptions are attributed to the Greek Hippocrates, the ‘Father’ of Medicine (≈460–370 BC), to whom the analogy between malignancies and ‘crab’ (with which a Greek wording for cancer, *carcinos*, is related), due to the projection-like outgrowths of the disease resembling the decapod crustaceans’ locomotory appendages. The Greek physician Galen (130–200 AD), practicing in Rome, first used the term *oncos* (‘growth’) to describe cancer, which stands today as the prefix of cancer-related terms such as oncogenesis (the complex process by which normal cells transform into cancer cells). Nonetheless, the oldest medical mentions to cancer date back to at least 1600 BC, even though they are likely based on information collected up to a thousand years before, if not more. They were found in ancient Egyptian writing (the ‘Edwin Smith’ and ‘George Ebers’ papyri) that, inclusively, already mentioned treatment by surgery, pharmacology and magic rituals [1,2].

Today, cancer is acknowledged as the principal cause of mortality worldwide, being responsible for about 10 million fatalities annually (with about twice as many new cases per year), figures that, led by in cases and death rate by lung cancer, can only partly be explained by population growth or socioeconomical development, as they may double in the next decade [3]. Even though the exact proportion of chemical-induced cancer is extremely difficult to estimate, Colditz and Wei [4] estimated that, excluding tobacco smoking, it may attain 10%. The same authors also noted that, due to legislation reducing occupational exposure for workers of high-risk industries (such as steel mills) in developed countries from late 20th century onwards, risk can actually be underestimated globally, since a great deal of heavy industry has been transferred to less-developed countries, where cases are likely underreported. Altogether, the incidence of cancer driven or at least co-adjuvated by exposure to pollutants represents a very significant portion of all cases and is seemingly increasing. As suggested by the European Commission Joint Research Centre (EC-JRC), “*Public health policy actions cannot be decoupled from environmental policy actions, since exposure to chemicals through air, soil, water and food can contribute to cancer and other chronic diseases*” (sic Madia et al. [5]). Needless to say, the challenges of describing and mitigating human health risks are but a fraction of those related to ecosystem and wildlife. Considering that malignancies have been found in 70-million-year-old dinosaur fossils [6], it is safe to assume that cancer is transversal at least among vertebrates, which renders environmental carcinogens not just a key problem for environmental toxicologists in general, but also to ecotoxicologists.

Cancer is essentially a genetic disease. Despite its long record in human medicine, the causes of cancer only began to be understood in the 20th century, especially after the discovery of the role of DNA and its chemistry; first in 1944 with the discovery by Avery et al. [7] that DNA is the ‘transforming principle’ in heredity; then with the famous ‘photo 51′ of Franklin and Gosling [8], which was paramount to the double helix of Watson and Crick [9]. Interestingly, the first human oncogene was only described in the early 1980s [10], following the pioneer work of Duesberg and Vogt [11] on avian retroviruses and transforming factors leading to sarcomas (malignancies of mesodermal-origin tissues, such as sub-dermal connective tissue). Almost immediately, Reddy et al. [12] disclosed molecular events underneath proto-oncogene activation into full oncogene, disclosing that all it may take is a single-point mutation. Quite naturally, this raised important questions among genotoxicologists, as it provided important insights on the mechanisms by which pollutants are oncogenic, even though we know today that different toxicants imply distinct modes-of-action, an issue that will be addressed further on. In the aftermath of these discoveries, just before the end of the 20th century, toxicologists and oncobiologists discovered that certain toxicants can cause specific mutations to oncogenes, leading to up-regulation of oncoproteins and therefore unbalancing cell cycle in favour of proliferation with prejudice of DNA checkpoints, cell cycle arrest and apoptosis; or, conversely, hindering the expression of tumour suppressors such as TP53. One of the most significant discoveries relates to polycyclic aromatic hydrocarbons (PAHs), many of which yield mutagenic metabolites (such as epoxides and some quinones) following phase I bioactivation by CYPs [13]. We now know that the formation of bulky DNA-metabolite adducts is not stochastic. Instead, there are preferential binding sites in specific regions of proto-oncogenes, such as those of the human ras family, promoting mutation due to problems in the removal of these lesions that lead to the conversion of the genes in oncogenes. increasing expression and subsequent up-regulation of oncoproteins (see Ross and Nesnow [14]). Considering the prevalence of PAHs in emissions such as tobacco smoke plus fumes and their particulate matter, this issue has been receiving specific attention due to its implications in human lung cancer (reviewed by Moorthy et al. [15], DeMarini and Linak [16]). The paradigmatic case study of PAH mutagenicity and carcinogenicity illustrates that cell and molecular biology laid important foundations for establishing causation and both human and ecological risk assessment. However, there are other fields of research that revolutionised toxicology and must thus not be forgotten. It is the case of the teachings of the British mathematician turned biologist Ronald Fisher (1890–1962), which, despite being involved mostly in population genetics (he was a polemic advocate of eugenics and endorsed human race distinction), arguably constructed the basis of modern statistics and biostatistics that are paramount for toxicologists and that are in the core of computational biology methods. Similar acknowledgements must be given to epidemiology, environmental chemistry and ecology.

It must be noted that pollutant-induced carcinogenesis in humans is just a small part of a large environmental problem. Even though the nature and function of detoxification phase I/II enzymatic systems and their role in neoplasia in non-vertebrate animals is far from consensual, in large part due to unstable substrate-dependent evolution from common ancestry [17,18], they tend to be well-conserved amongst vertebrates, including fish, which sustains that chemical mutagenesis and oncogenesis are also a serious ecotoxicological problem. In fact, ecotoxicologists have been in the frontline of research and are responsible for major developments in risk assessment, biomarker plus bioassay development and toxicogenomics. This contribution owes much to the fact that ecotoxicologists have to deal with challenges like toxicant mixtures in intricate matrices (such as natural sediments); unconventional model species with reduced or absent genomic annotation and unaccountable noise variables compromising causation [13,19,20].

Altogether, toxicology followed a long path since the observations of Paracelsus on lung diseases of miners, but centuries later even abolishing asbestos seems retrospectively to have been a rather long an obstacle-ridden process from causation to legislation. How can these situations be avoided in the future now that we analytical and computational tools to look for entire molecular pathways in single runs? Rather than an extensive appraisal of the incidence of chemically induced cancer under a biomedical and epidemiological perspective, for which there are thorough discussions elsewhere, the current work is a short review directed especially to toxicologists. Most especially directed at establishing a bridge between environmental toxicologists and oncobiologists, an association that is not often explored in specialty reviews. Here, we will summarise the history and state of the art of research and its methods in the field; hopefully a ‘heads up’ to increase awareness for the many challenges ahead in terms of both human health risk assessment and ecosystem monitoring for the causes and prevention of what is rightfully named the epidemic of 21st century.

## 2. A Brief Historical Overview of Cancer and Pollution

**Figure 1:**
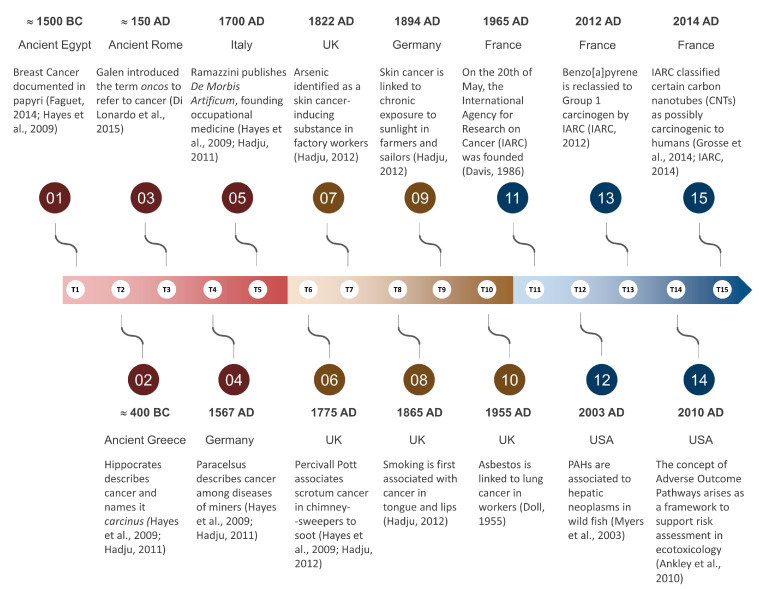
Important milestones in ‘oncotoxicology’, from the first written records to modern days. Main sources [2,19,21,23,24,25,26,27,28,29,30,31,32] are indicated as per reference list.

The great scientific gap of the Dark Ages brought biomedical sciences in western civilisation to a stand-still. However, Arabic physicians like Avicenna (980–1037) and philosophers like Averroes (1126–1198) collected, expanded and taught scientific knowledge, essentially keeping alive the flame of early biomedicine in times of strict Christian fundamentalism and feudal obscurantism. Their work and teachings were fundamental pillars upon which European medicine would be rebuilt during the Renaissance. The Swiss-German physician and alchemist Theophrast von Hohenheim (1493–1541), better known as Paracelsus, who is considered the founder of modern toxicology by first acknowledging dose-effect relationships (‘it is only the dose which separates benefit from poison’), was seemingly the first to draw conclusions on chemical-induced carcinogenesis. In his posthumously published work on the ‘mountain disease’ of miners (1567), Paracelsus already linked illnesses such as tuberculosis and lung cancer to exposure to ‘poisonous air’. This work (originally named *Von der Bergsucht und anderen Bergkrankheiten*) was born from Paracelsus’ direct observations of miners and their environment. It can very well be the first occurrence of what we would nowadays call an association between occupational exposure and cancer—in a time were diseases were mostly attributed to mystical causes (the reader is diverted to the review by Hayes and Gilbert [21] on the hallmarks of toxicology). Additionally, the Italian physician Bernardino Ramazzini (1633–1714), often regarded to be the founder of occupational medicine, in his most famous book, *De Morbis Artificum Diatriba* (‘On the Diseases of Workers’), already discussed the environment-related aetiology of various diseases, from pneumoconiosis to breast cancer (see Franco [22]). It needs to be noted, though, that if Theophrast established a link between toxicology and disease and Ramazzini associated occupational exposure to disease, including cancer, the study of chemical toxicology at this stage was still struggling with establishing causation due to the lack of identification of carcinogens *per se*. Nonetheless, despite rudimentary or *a priori* absent tools, The Illuminist Man would slowly, but steadily, find his course (see Figure 1 for a historical overview).

In the times that preceded the Industrial Revolution, the English physician Percivall Pott (1714–1788), building on the work of Paracelsus, associated testicular squamous cell carcinoma, a typical scrotum malignancy with chimney sweepers and exposure to soot. This can effectively be regarded as the first time the carcinogenicity of pyrogenic (combustion-derived) polycyclic aromatic hydrocarbons (PAHs) was documented, even though it would take about 200 years to understand the chemical nature of carcinogens in tars. In fact, only in the 1920s were PAHs identified as the carcinogenic agents in coal tars, with emphasis on the (in)famous benzo[a]pyrene [33]. Such discoveries followed a series of experiments with murines ultimately led by the British pathologist Ernest Kennaway but based on the works on skin cancer by two outstanding Japanese pathologists, Katsusaburo Yamagiwa and his assistant Koichi Ichikawa [34], who are effectively accredited for the discovery of environmental chemical carcinogenesis in the aftermath of their experiments on chemically induced cancer by painting tars on the ears of rabbits. These pioneering works are directly aligned with the reports on the prevalence of skin cancers among workers handling paraffins, petroleum and tars dating from the previous century [35,36]. These examples illustrate the difficulties in establishing causation even well after the First Industrial Revolution (late 1700s to late 1800s). In large part, this is due to the lack of a well-defined experimental method (including statistics) in life sciences and the missing gaps in what we call today biology 101 that made it impossible for researchers to understand *why* cancer occurs, which is a paramount question that still puzzles present-day scientists. In fact, despite of the discovery of cells by Robert Hooke (1635–1703), published in 1665 in his book ‘*Micrographia*’, only in the mid-19th century was Cell Theory effectively established as one of the dogmas of Biology by the hands of three German scientists, Theodor Schwann, Matthias Jakob Schleiden and Rudolf Virchow, who, nevertheless, worked autonomously. The 19th century also witnessed the revelation of evolution and natural selection through Darwin, Wallace and Haeckel; Mendelian heredity and DNA itself in 1869 (as ‘nuclein’) by the Swiss chemist Friedrich Miescher (1844–1895). It would require a century before these three hallmarks of biology would be pieced together in a unified theory, though. This means that the mechanistic perception of cancer as a genetic disease with origin in mutations, hereditary or acquired, would have to wait more than 100 years to mature. Nonetheless, in 1866, the eminent French physician Pierre Broca (1824–1880) had already noted hereditary predisposition for breast cancer in some families. Additionally, the Nobel prize-winning US pathologist Francis Peyton Rous (1979–1970) identified for the first time that a pathogen (a retrovirus infecting chickens) could cause cancer (the Rous Sarcoma that would later be fundamental for the abovementioned discovery of oncogenes).

Also of great importance is the link between the formation of micronuclei and neoplasic cells by the German cytologist Theodor Boveri (1862–1915) from his work with echinoderm embryos [37]. Micronuclei and other nuclear abnormalities are, to date, acknowledged biomarkers of malignancies and of genotoxicity (damage at whole-chromosome level, in the case), spanning from humans to wildlife [38,39,40]. Together with the alkaline Comet assay method for the detection DNA lesions at the strand levels [41], the quantification of nuclear abnormalities in peripheral cells (including the haemocytes of invertebrates) is still one of the most important and expeditious biomarkers for genotoxicants deployed by environmental toxicologists and ecotoxicologists. The first-time micronuclei were associated with exposure to an environmental agent (ionising radiation, in the case) occurred in 1959 [42]. Interestingly, this happened well after the discovery of X-ray-induced DNA damage and mutagenesis in *Drosophila* in the 1920s [43]. It also followed the anecdotal case of the wealthy US golf amateur champion and socialite Eben Byers (1880–1932), who had a gruesome death after being devoured by mouth and gut cancer (not radiation poisoning as reported at the time), after years of drinking radioactive water, a patented tonic (‘Radithor’) that was advertised as an endocrine stimulator by the famous charlatan William Bayley in a time when selling radioactive pseudo-medicines, cosmetics and materials was common practice, without neglecting the early amazement and overuse of X-ray medical imaging. We may also recall the sad case of the ‘radium girls’ of the 1920s, US workers that suffered from all sorts of radiation-related sickness acquired from watch dial ‘self-luminous’ radioactive paints [44].

The end of World War II unleashed an era of technological marvel that, just like the Industrial Revolution(s) before it, was responsible for the mass production of old and novel chemicals. However, as previously mentioned, neither environmental quality not occupational medicine were mainstream issues. The status quo would change with a series of events starting in the 1960s that demystified that the environment was an infinite entity that could absorb all of our waste and dilute it to safe levels. Rachel Carson denounced pollution by pesticides, especially organochlorines like DDT (dichloro-diphenyl-trichloroethane), which was developed in the 1940s and the first modern, safe, synthetic insecticide [45], and shortly after Clair Patterson did the same on the catastrophic global contamination by lead from fuel additives [46]. Even though it would take decades to see any effective consequences for Carson’s and Patterson’s invaluable work, the United Nations Stockholm Convention of 2001 imposed a worldwide ban on DDT (with few emergency exceptions related to the control of insects as disease vectors), whereas Algeria was the last country in the world to stop commercialising leaded gasoline, in 2021. Importantly, it must be noted that DDT is nowadays considered by the International Agency for Research on Cancer (IARC) as ‘probably carcinogenic to humans’ (Group 2A), similarly to inorganic forms of the highly toxic metal lead (Pb), based on sufficient evidence on experimental animals, even if not from humans [47,48]. These case studies illustrate perfectly challenges met by scientists, particularly the fierce opposition they face when environmental and human health collide with major economic factors.

Altogether, the 1960s and 1970s formed an era of very active social and environmental activism that, regardless of the actual speed of implementation of policy and guidelines, led to profound societal changes. Besides bans and limitations on specific substances (some which will be addressed in a subsequent section), IARC itself was founded in 1965; the U.S. National Oceanic and Atmospheric Administration (NOAA) National Status and Trends (NS&T) Mussel Watch Program started in 1986 and is the oldest running marine biomonitoring programme in the world, focusing on more than 100 priority pollutants, including many environmental carcinogens; the implementation of the International Chemical Safety Cards (ICSCs) by the World Health Organisation (WHO) and the International Labour Organisation (ILO) as a tool to protect workers’ safety, or the London Convention of 1972, which aimed at prohibiting the dumping of hazardous waste in the oceans, military or civilian (signed by just 90 countries to the present day, though), to quote a few examples. Still, despite such measures, the first compelling causal relationship between PAH pollution and tumours in marine fish in their native environment was only established in the early 21st century [29]. In another example, the potential carcinogenicity of certain multi-walled carbon nanotubes (MWCNTs) was only acknowledged by IARC [31,32] just 23 years after the unravelling of CNTs [49], hitherto motivated by the growing interest in nanotechnology. These two instances illustrate not only the rapid advance of toxicological sciences but also awareness for cancer-inducing agents. Nonetheless, it is clear that there is still a considerable time gap between the introduction of a new chemical (or nanomaterial) and acknowledging its risks, which is yet another challenge that must urgently be resolved. In fact, twenty-three years may be seen as short period, but it effectively represents the span of a generation. And it took only the time of a generation to witness the rapid expansion of quack radioactive pseudo-medicines and cosmetics between 1920s and the 1950s. Even though our current level of knowledge and, moreover, risk assessment policies and guidelines (which emerged from the synergy between science and public awareness more than from goodwill of political or economic leadership) greatly diminishes the possibility of these events being repeated, they are not unlikely to happen given the impressive variety and quantity of new compounds that are introduced in the market yearly. The combined effect of legacy (traditional) and novel (emerging) pollutants on humans and ecosystems will be ascertained in the near future and our role in risk assessment and mitigation will be judged by future generations.

## 3. A Traditionalist View on Legacy and Emerging Carcinogens

The combined number of compounds nowadays listed by IARC as carcinogenic to humans (Group 1), plus *probably* and *possibly* carcinogenic to humans (Groups 2A and 2B, respectively), now exceeds 500, out of 1000 assessed individual compounds and a few mixtures like exhaust fumes and bitumen. Note that Group 2B differs from 2A by further limitations on available evidence from humans and experimental animals, albeit still supported by at least strong mechanistic evidence. This list of classifications is an invaluable resource; however, it does not account for all causative agents for common human cancers. At this point, the reader may refer to Cogliano et al. [50], for a review on IARC classifications and their importance for biomedicine. This list includes several compounds that can be considered historical landmarks for environmental toxicologists (i.e., those with closer interest on the human-environment interface) and ecotoxicologists. Here we will briefly review a few notorious case studies that stand out due to factors such as historical relevance or mode-of-action.

### 3.1. The Paradigmatic Case of Asbestos

Asbestos is arguably one of the most paradigmatic cases of environmental carcinogens. Asbestos is a generic name for a group of six different silicate minerals that became highly valuable in heavy industry and construction because they form fibres that are resistant to heat, oxidation and electrical conductivity, thus offering a simple and cheap insulant. For such reason, from the late 19th to the late 20th centuries, the world would witness a long struggle between the powerful asbestos lobby and workers, which were ultimately supported by legislators, especially in France and subsequently the EU.

Ever since Paracelsus’ observations on ‘mountain disease’, miners and other workers of related industry have been a case-study for occupational exposure to carcinogens. Asbestos, the cheap ‘miracle’ fibre that could be mixed with concrete and other material to protect structures from fire and corrosion, has actually been associated with lung disease since the late 19th century, but it would take more than 50 years to build a solid body of evidence that could be put against industrialists and lobbyists. In the 1950s, Doll used statistics to associate lung cancer with occupational exposure to asbestos amongst miners in the UK [27]. However, twenty years earlier, Vorwald and Karr [51] had already investigated a potential relationship between dust-caused lung disease (pneumoconiosis) and lung cancer in miners, including a pleural malignancy named mesothelioma that seems to be exclusively caused by exposure to this mineral. In fact, asbestosis had already been serious among workers since the late 1900s, but it took a major conference organised by the New York Academy of Sciences in 1964 on asbestos and its hazards to make the general public aware of the problem, despite hundreds of thousands of asbestos workers developing lethal sickness following the inhalation of asbestos nanofibers [52]. The case hitherto built against the use of asbestos would become groundbreaker for nanotoxicologists as well. It must be noted that nanomaterials are such a particular case that they deserve a toxicology branch of their own, because nanoparticles (particles less than 100 nm in at least of its dimensions) behave differently from chemicals and materials. Interestingly, even today the mechanistics underlying for formation of mesotheliomas is not consensual and it is likely that there is not a single pathway acting on its own and may involve oxidative stress, inflammation or interference with the tissues’ proteome [53].

### 3.2. Arsenic, the Base of the First True Medicine

**Figure 2:**
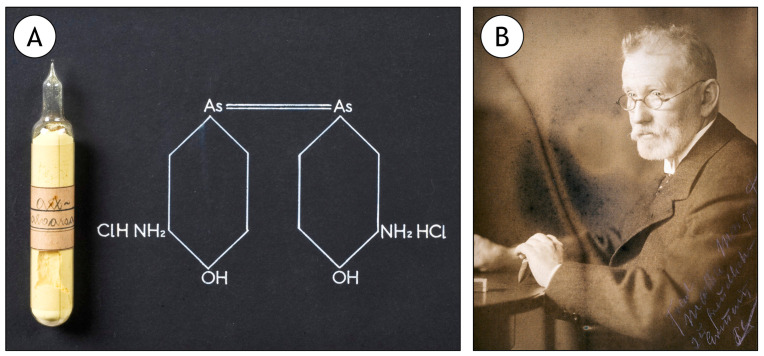
The first true biomedical drug (1910) was an anti-syphilitic drug based on the environmental carcinogen As. (**A**) Surviving original ampoule and formula of Salvarsan (Arsphenamine). (**B**) Salvarsan’s inventor, Paul Ehrlich (1854–1915). Both images were retrieved from the public domain and are subject to a Creative Commons Attribution (CC BY 4.0) licence. Credit to the Science Museum (London) and the Wellcome Collection (London) for panels A and B, respectively.

The metalloid arsenic (As) and its inorganic forms are also classified by IARC as carcinogenic to humans (Group 1) [54]. Its historical conflict between environmental hazard and medicinal use makes it a particular interesting case-study. In fact, we must not forget that Paul Ehrlich’s Salvarsan (arsphenamine) of 1910, considered to be the first true pharmacological drug for being specifically conceived to target syphilis, was arsenic-based (Figure 2). Arsenite (As^III^) is the most toxic form of the metalloid that is a ubiquitous environmental contaminant with both anthropogenic (such as the production and use of fertilisers and smoking) and natural sources (mainly from the hydrodynamic erosion of rocks). As such, besides a major problem for human health, As is a serious ecotoxicological problem, especially in aquatic ecosystems, even though As-driven oncogenesis in non-model organisms is little studied. Contamination can occur via air, water and even food, as it is easily bioaccumulated and even biomagnified along the food chain; altogether being associated to cancer of the skin and lung, especially. Arsenic can interfere with many molecular processes, which means, once again, an intricate mode-of-action that includes interference with redox processes in the cells, altering epigenetics and genotoxicity [55,56].

### 3.3. Cadmium versus PAHs: A Matter of Mechanism

In general terms, the carcinogenicity of metals is diverse and tend to depend on species and form (organic vs. inorganic). For instance, organic mercury (methylmercury), which is a potent toxicant for humans and wildlife, is *only* classified Group 2B whereas the metallic form is Group 3 (‘not classifiable as to their carcinogenicity to humans’). One of the most toxic metallic pollutants, cadmium (Cd) is, nonetheless, classified as Group 1 [57]. Cadmium is a non-essential element first reported as highly toxic in the mid-19th century, specifically following the use of cadmium carbonate powder as a polishing agent, which resulted in gastrointestinal and pulmonary problems [58]. This toxic metal is a fairly common pollutant, especially in aquatic ecosystems impacted by industry. Contamination results from a broad range of applications, from metal alloys and amalgams (dental amalgams in the past, inclusively) to paints and batteries. What makes the metal’s carcinogenicity particularly interesting is its mechanistics. Cadmium is not a Fenton metal and, therefore, does not generate oxidative radicals *per se*. Additionally, it can only enter the nucleus and intercalate with DNA in extremely high doses; however, it can replace essential divalent ions (such as Zn^2+^ and Ca^2+^) from the active centre of enzymes, with emphasis on DNA repair enzymes, to which is added inhibition of apoptosis [59,60]. The metal is therefore an indirect genotoxicant and mutagen by enabling the accumulation of either naturally occurring DNA lesions or lesions caused by other compounds, which raises important questions on chronic exposure to mixed toxicants, which is actually the most likely scenario in any anthropogenically-impacted area.

Cadmium is a model (legacy) pollutant for aquatic ecotoxicologists. Its aforesaid mode-of-action on the impairment of DNA repair (and other metabolic pathways, from oxidative stress to endocrine disruption) is known to be shared at least with fish (see Kienzler [61] and Liu et al. [62]). Its genotoxic effects at strand and chromosomal levels (micronuclei induction) are known even in the haemocytes of bivalves [63]. However, as for the vast majority on pollutants, specific studies of neoplasms in wildlife are virtually absent. Interestingly, Cd shares its historical late acknowledgement as carcinogenic to humans with another interesting legacy toxicant: the PAH benzo[a]pyrene. In fact, despite the well-known risks associated to PAHs, only benzo[a]pyrene is classified by IARC as Group 1 (‘carcinogenic to humans’), a classification that dates only from 2012 despite consideration since early 1973s [30]. Nonetheless, it must be emphasised that this information is, on its own, misleading, since it is explained by constraints in gathering sufficient epidemiological data despite the good amount of experimental data retrieved from animal testing.

### 3.4. Novel and ‘Emerging’ Pollutants

Among substances of emerging concern, we may find very distinct agents, from human and veterinarian pharmaceuticals to novel pesticides and nanomaterials, for instance (the carcinogenic potential of MWCNT has been discussed above). A notorious case is glyphosate, a synthetic compound designed to target the shikimate pathway of weeds most commonly commercialised as the formulation called ‘roundup’. This compound is one of the most widely used herbicide globally and has recently been reclassified by IARC as ‘probably carcinogenic to humans’ (therefore pertaining to Group 2A), based on limited but sufficient evidence from experimental animals [64]. Glyphosate, which has been advertised as ‘safe’ has been thoroughly investigated by toxicologists, especially ecotoxicologists, but the results are often unclear or contradictory; for instance, with respect to genotoxicity (for a recent review see Ojelade et al. [65]). However, statistics suggest that glyphosate-based herbicides can increase the risk of non-Hodgkin lymphoma (NHL) in humans by 40% [66].

In turn, data on pharmaceuticals and their metabolites are comparatively scarce, even though they are some of the most important emerging pollutants, not only due to the growing use and diversity of compounds but also because their removal from wastewater tends to be inefficient [67]. Still, an interesting case is that of the ‘conjugated oestrogen therapy’ for menopause, a mixture of synthetic hormones that has been linked to breast cancer. Its classification has been revised as Group 1 [68], despite consideration since the 1970s, when the association between oestrogens and endometrial cancer was first suggested [69]. Considering the importance of endocrine disruptors for humans and wildlife, further investigations on the effects and modes-of-action of these emerging pollutants, carcinogenic or other should perhaps deserve further attention from researchers and policymakers.

## 4. Novel Approaches to Mechanism and Causation: Cancer and the Exposome

For a toxicologist, the integration between exposure, toxicity and mechanism should ultimately provide a reliable measure of risk for both humans and wildlife. In this respect, cancer can be regarded as any other pathological outcome from exposure to noxious agents, in spite of the complexity of cellular and molecular processes underlying oncogenesis that, in turn, are shaped by unsurmountable internal and environmental variables, including toxicants. This implies pushing toxicological risk assessment toward more holistic approaches. One of the most paradigmatic changes in life science research that would be welcomed by toxicologists was the Systems Biology framework introduced by Ideker et al. [70,71], which fundamentally proposes that an integrated assessment of a systematically perturbed biological system at various levels of organisation can be used to build predictive models of its functioning. Sturla et al. [72] essentially adapted the Systems Biology conceptual framework into toxicology, since the building of holistic and quantitative models of biological systems, that can predict the outcomes when the same system is challenged by toxicants, seemed a perfect match to the purpose of toxicological risk assessment.

Systems Toxicology expanded environmental toxicology beyond the narrow single-endpoint biomarker concept. Since it is now acknowledged that exposure to noxious agents affects entire molecular pathways and gene networks, potentially leading to highly complex pathological traits downstream, Ankley et al. [19] introduced the concept of Adverse Outcome Pathway (AOP), which can be defined as a series of interlinked events as various levels of biological organisation triggered by a given stressor, as an approach to risk assessment in ecotoxicology that other domains of toxicology are now incorporating as well. This development shows that ecotoxicologists must not be forgotten as pioneers in toxicological sciences and that important lessons can be retrieved from those accustomed to dealing with complex scenarios.

In brief, an AOP implies binding a molecular initiating event (MIE) to a given adverse outcome (AO) via different key events (KE) that articulate through key event relationships (KERs). In our particular case, the AO would naturally be, of course, cancer. Quite naturally, finding AOPs implies systematising the search for genes and their networks, proteins or metabolites, which is greatly supported by ‘omics’ methods that revolutionised toxicogenomics, with emphasis on transcriptomics and proteomics, unavoidably supported by bioinformatics and the rapid expansion for public-access databases for ‘omes’, molecular pathways, protein-protein interactions and others [19,73,74]. Despite the enormous challenges and potential pitfalls ahead, environmental toxicologists and, moreover, ecotoxicologists, for whom the ability to assemble proteomes and transcriptomes *de novo* (without mapping) enabled by advances in mass spectrometry and next-generation sequencing of RNAs (RNA-Seq) offered unique possibilities to study non-conventional models, swiftly adhered to these methodologies.

The Organisation for Economic Co-operation and Development (OECD), whose relevance for setting the standard for chemical safety exceeds its signatories, swiftly adopted AOPs with the ultimate aim at building a comprehensive database, for chemically induced cancer, inclusively. As an example, an AOP for hepatic cancer involving CYP2E activation (a monooxygenase involved in the bioactivation of dozens of low-molecular weight toxicants, from ethanol and acetone to some pharmaceutical drugs) has been recently published [75]. In another example, Nymark et al. [76] proposed a AOP for lung cancer based on eighteen standardised tests especially developed for the risk assessment of nanomaterials. In addition, Benoit et al. [77] proposed an AOP for breast cancer (i.e., the AO in the case) whose MIE is the activation of the AhR (aryl hydrocarbon receptor), a pivotal molecule in exposure to many carcinogenic compounds, including PAHs. An important advantage of finding AOPs is that they may circumvent the lack of a comprehensive view of an organisms’ exposome for the purpose of risk assessment. The concept of ‘exposome’ was first introduced by Wild [78] in the aftermath of the sequencing of the human genome to remind us that a human being is more than a mere map of genes and refers to all external (environmental) and internal (endogenous) factors that can ‘shape’ an organism throughout its life. Under a toxicologist’s point-of-view, understanding the exposome should mean establishing causality, however, discovering even the main agents that an organism has been exposed to at some stage of its life can seem unsurmountable. Without prejudice for the need to isolate causative agents, associating MIEs (the molecular trigger in the cell) to an AO (the effect) may provide a measure of risk (i.e., a *probability* of harm) without *a priori* complete knowledge on the exposome.

## 5. Conclusions

The ‘One Health’ approach upheld by the World Health Organisation (WHO) is the ultimate acknowledgement that environmental health means human health. It took us centuries to celebrate the first solid associations between chemicals and cancer and, in the early 21st century, the number of confirmed environmental carcinogens may seem disappointing in face of the growing diversity of novel pollutants and the spectre of estimating risk for complex mixtures of noxious agents. However, from molecular biology to statistics and computation, we mustered an impressive set of tools since the discovery of the role of DNA in cells about a century ago. Additionally, social awareness for environmental problems grew exponentially in the last decades. One of the most important lessons is that many technical advances have actually been introduced or adapted by ecotoxicologists as means to address extremely complex scenarios. It can thus be rightfully expected that synergies between ‘eco’ and ‘human’ toxicology will continue to develop in the future. Another important lesson is the growing awareness that mechanism is essential to determine risk. Understanding how cancer occurs provides biological plausibility and possibility of an adverse outcome when evidence for association between a specific chemical and neoplasia is difficult to achieve. Consequently, mechanism has a pivotal role in the shortening of the time gap sapping between the development and release of a new potentially carcinogenic agent and the implementation of safety and mitigation measures. Altogether, investigating Adverse Outcome Pathways for various cancer types can thus produce an invaluable resource not only to rapidly predict carcinogenic risk for novel chemicals and materials in complex contamination scenarios for humans and wildlife.

## Data Availability

Not applicable.

## Abbreviations

Ahr	aryl hydrocarbon receptor
AO	adverse outcome
AOP	adverse outcome pathway
B[a]P	Benzo[a]pyrene
CYP	cytochrome P450
DDT	dichlorodiphenyltrichloroethane
IARC	International Agency for Research on Cancer
KE	key event
KER	key event relationships
MIE	molecular initiating event
MWCNT	multi-walled carbon nanotube
NP	nanoparticle
OECD	Organisation for Economic Co-operation and Development
PAH	polycyclic aromatic hydrocarbon
WHO	World Health Organisation
